# Sex/Gender Differences in the Effects of Childhood Abuse on Future Risk of Depression: Longitudinal Evidence from the Global Flourishing Study

**DOI:** 10.3390/children12060754

**Published:** 2025-06-11

**Authors:** Shervin Assari, Babak Najand, Alexandra Donovan

**Affiliations:** 1 Department of Internal Medicine, Charles R Drew University of Medicine and Science, Los Angeles, CA 90059, USA; alexandradonovan@cdrewu.edu; 2 Department of Psychiatry, Charles R Drew University of Medicine and Science, Los Angeles, CA 90059, USA; 3 Department of Family Medicine, Charles R Drew University of Medicine and Science, Los Angeles, CA 90059, USA; 4 Department of Urban Public Health, Charles R Drew University of Medicine and Science, Los Angeles, CA 90059, USA; 5 Marginalization Related Diminished Returns Center, Los Angeles, CA 90059, USA; babaknajand@diminishedreturns.org

**Keywords:** adverse childhood experiences, depression, gender differences, global flourishing study, childhood abuse, longitudinal study

## Abstract

**Background:** Adverse childhood experiences (ACEs) such as abuse (defined as emotional, physical, or sexual abuse without distinguishing type or severity) have long been linked to mental health challenges in adulthood. However, less is known about how these effects differ by sex/gender in global samples, particularly using large-scale, multi-country panel data. **Objectives:** To examine whether the long-term association between childhood abuse and changes in depressive symptoms during adulthood differ between men and women, after adjusting for demographic and socioeconomic factors. **Methods:** We conducted a secondary analysis of Waves 1 and 2 of the Global Flourishing Study (GFS), a longitudinal panel study covering 22 diverse countries. The sample included adult participants with complete data on childhood abuse (yes/no); depression at baseline and follow-up; sex/gender; and relevant covariates (age; education; marital status; immigration status; smoking status; and employment). Depression was measured using a two-item scale. Multi-group structural equation models were used to test the effect of ACE on changes in depression over time where groups were defined based on sex/gender. **Results:** Overall, childhood abuse was associated with a statistically significant increase in depression scores between Wave 1 and Wave 2. This association was significant among women but not among men. **Conclusions:** Our findings suggest that the mental health consequences of childhood abuse extend into adulthood and disproportionately affect women. These sex/gender differences may reflect variations in stress processing; coping; and social roles. Interventions addressing early adversity may need to be tailored to recognize and respond to such sex/gender-specific vulnerabilities.

## 1. Introduction

Depression is more common among women and girls than men and boys [1], and various biopsychosocial underlying mechanisms have been proposed to explain this disparity, ranging from hormonal differences (sex differences) to patterns of stress exposure (gender differences) [2]. For example, the COVID-19 pandemic has led to increased rates of depression and anxiety, particularly among women. One review explored the underlying contributors to these sex differences, discussing transcriptomic and genetic factors, neuroendocrine regulation, immune function, and cognitive processes. The authors also examined sex differences in antidepressant response and their potential biological underpinnings. They emphasized the critical importance of integrating sex as a biological variable in both preclinical and clinical studies to facilitate the discovery of more effective pharmacotherapies for all genders [3].

Adverse childhood experiences (ACEs) have been consistently linked to poor mental health outcomes in adulthood, particularly depression [4,5]. Experience of abuse during childhood can disrupt developmental and emotional trajectories and increase vulnerability to psychopathology [6,7]. However, growing evidence suggests that these effects are not uniform across population subgroups, particularly those based on sex and gender. One hypothesis suggests that females may be more susceptible to stressors such as adverse childhood experiences (ACEs), including child abuse (defined as emotional, physical, or sexual abuse without distinguishing type or severity), compared to males [8]. It has been proposed that women are more likely to be risk-averse [9] and ruminate in response to stress or loss, which may increase their vulnerability to the effects of ACEs on depression risk [10,11]. According to this perspective, even at the same level of stress exposure, psychological and emotional outcomes such as depression may be more severe for women and girls than for men and boys [11].

Numerous review articles [3,12] and empirical studies [13,14] have examined sex and gender differences in depression. One review emphasized that the adverse consequences of ACEs on adult outcomes are influenced by biological sex, the developmental period during which stress occurs, and the life stage at which outcomes are assessed. The authors highlighted that sex differences in adult depression may be shaped by various mechanisms, including sex hormones, serotonergic pathways (5-HT), the hypothalamic–pituitary–adrenal (HPA) axis, and epigenetic modifications [15]. Findings from epidemiological research document sex-based disparities in the onset, prevalence, and clinical features of depression. While social and behavioral factors contribute to these disparities, several biological systems also appear to play a central role. These include sex-specific brain structures and neural circuitry, reproductive hormones, stress reactivity, immune responses, metabolism, and patterns of fat distribution. The authors argued that reproductive transitions unique to women—such as menstruation, pregnancy, postpartum, and menopause—represent windows of heightened psychological vulnerability. Additional biologically rooted risk factors, including sleep disturbances and exposure to early-life trauma, may also contribute to sex differences in depressive symptoms [16].

Several empirical studies further support these observations. One study using data from the National Longitudinal Study of Adolescent to Adult Health assessed the effects of 10 ACEs on C-reactive protein (CRP) and depressive symptoms in adulthood. Interaction terms and sex-stratified models were used to evaluate sex differences. Emotional abuse and parental incarceration were consistently associated with both CRP and depressive symptoms in both sexes, but other associations were sex-specific. For instance, childhood maltreatment was more strongly linked to depressive symptoms in females, while sexual abuse was more closely associated with inflammation in males. The authors highlighted the importance of considering how ACEs cluster differently by sex and how these differences inform both theory and intervention design [17,18,19,20,21]. In another study involving 826 adults (69.4% women), participants completed self-report measures of stress, psychological abuse, depression, and anxiety. Psychological violence was found to mediate the relationship between recent stress and both depression and anxiety. Importantly, sex moderated these associations, suggesting that interventions should be sensitive to gendered experiences of stress and violence to avoid reinforcing biases in clinical practice [22]. Finally, a psychometric network analysis of data from the Midlife Development in the United States study (N = 1917) examined associations among childhood trauma, major depression, and inflammation markers. Separate network models for men and women revealed both shared and sex-specific associations. Emotional abuse was strongly linked to somatic symptoms, and notable differences emerged in how inflammation and depressive symptom clusters were interconnected across sexes. These findings suggest that sex-disaggregated network models may identify distinct pathways and treatment targets for men and women [13].

Donovan and colleagues have proposed a conceptual framework suggesting that exposure to ACEs such as childhood abuse may influence the risk of depression and substance use differently in males and females [23,24]. Their literature review and theoretical framework highlights biological, psychological, and social factors which may contribute to these disparities. They hypothesize that hormonal differences during puberty and sociocultural influences including gender norms may disproportionately increase women’s rather than men’s vulnerability to the effects of exposure to ACEs on depression and substance use. ACEs may lead to greater hormonal dysregulation in females, potentially increasing the risk of internalizing symptoms and substance use during adolescence and beyond.

If there exists considerable sex and gender difference in the contribution of exposure to ACEs on depression, then interventions addressing ACEs and their consequences may need to be tailored to account for sex/gender-specific pathways [25,26]. Such tailored and sex/gender-specific interventions should consider both biological and sociocultural factors that differently influence the development of depression and substance use behaviors in males and females [27]. Among the moderating factors, sex and gender have emerged as key variables influencing the long-term impact of exposure to ACEs [23,24]. Biological, psychological, and sociocultural differences between men and women may shape their emotional responses, coping mechanisms, and access to support systems following exposure to early trauma. However, most existing studies on sex/gender differences in the mental health consequences of ACEs have been conducted in Western, high-income countries, limiting their generalizability. The Global Flourishing Study (GFS) [28,29,30,31,32,33] provides a unique opportunity to examine these associations in a large, diverse, and globally representative adult sample. This study leverages data from over 200,000 individuals across 23 countries, tracking various indicators of well-being over time, including exposure to childhood abuse (as predictor) and symptoms of depression (as outcome).

In this study, we test whether the association between exposure to childhood abuse and future changes in depressive symptoms during adulthood varies by sex/gender. In line with Donovan et al. [24], we hypothesize that the association between childhood abuse and future increases in depression is stronger among women than men [24]. We also control for demographic and socioeconomic variables that may confound or modify these associations. This study contributes to the literature by examining changes in depression over time, rather than cross-sectional associations, in a globally diverse cohort. The Global Flourishing Study’s multinational design, large sample size, and breadth of demographic representation offer unique insights into how the effects of early adversity on adult mental health may generalize across cultural contexts. Importantly, by linking childhood exposures to adult outcomes, the study underscores the long shadow that early trauma can cast well into midlife and beyond.

## 2. Methods

### 2.1. Design

This study used data from the Global Flourishing Study (GFS) [28,29,30,31,32,33], a large, longitudinal, multinational panel study that includes adult participants from 22 countries. The GFS was designed to examine factors related to well-being, mental health, and human flourishing across cultural and social contexts. Participants were surveyed at baseline and followed up approximately one year later, with information collected on demographics, socioeconomic indicators, past experiences of trauma, and mental health status, including depression.

Predictor: Our key exposure was self-reported history of childhood abuse, captured through retrospective items that asked whether participants had experienced emotional, physical, or sexual abuse in childhood. This was reported as a binary (yes/no) variable. The binary variable (yes/no) captures retrospective reports of emotional, physical, or sexual abuse but does not allow differentiation of type or severity, limiting dose–response analyses.

Outcome: The primary outcome was the change in depression over time, measured using a two-item validated depressive symptom scale administered at both baseline and follow-up [34]. Importantly, baseline depression scores were included as covariates in all models to assess relative change in depression rather than absolute levels, offering a longitudinal perspective on worsening mental health. The choice of a brief two-item scale was made to ensure feasibility in a multinational context and comparability across diverse cultures, though it may limit the full capture of the clinical spectrum of depression.

Confounders: Covariates included age, unemployment, marital status, immigration status, smoking status, and educational attainment. Education was categorized into three levels: low (e.g., no formal education or primary education only), mid (e.g., secondary education or some postsecondary), and high (e.g., college degree or above), with low as the reference category. We also controlled for country as fixed effects to account for demographic and contextual variation. All variables were standardized as needed to ensure comparability across countries.

Effect modifier: Sex/gender was the moderator of interest (1 for male and 0 for female).

### 2.2. Analysis

We employed structural equation modeling (SEM) [35,36,37,38] to estimate the effect of childhood abuse on depressive symptom change over time and to test whether this association varied by sex/gender. Multi-group SEM was used where groups were defined based on sex/gender. We did not fix any paths in our multi-group model [39]. SEM allowed for simultaneous modeling of measurement error and latent constructs while controlling for baseline depression and covariates [40]. A Root Mean Square Error of Approximation (RMSEA) value below 0.04 and a Comparative Fit Index (CFI) value above 0.95 were considered indicators of good model fit. Given the very large sample size, a significant chi-square test was not regarded as poor fit.

## 3. Results

Data from 124,982 participants across 22 countries were analyzed. On average, the time interval between the two measurement waves was 418 days (SE = 0.28). Table 1 presents the distribution of participants by country. The sample was not evenly distributed, with the largest proportion of participants from the United States (*n* = 31,970; 25.58%, 95% CI: 25.34–25.82), followed by Japan (*n* = 13,841; 11.07%, 95% CI: 10.90–11.25) and Sweden (*n* = 11,539; 9.23%, 95% CI: 9.07–9.39). Kenya (6.13%), Poland (5.14%), India (4.82%), and Tanzania (4.44%) also contributed substantially to the total sample.

Several other countries accounted for 2% to 4% of the sample, including China (3.63%), Brazil (3.36%), Germany (4.38%), and Egypt (2.42%). Countries with lower representation included Mexico (1.78%), South Africa (0.77%), Türkiye (0.40%), and Hong Kong (0.56%).

Overall, the dataset reflects considerable global diversity, with participants from Asia, Africa, Europe, North and South America, and Oceania. However, the unequal distribution of participants across countries should be considered when interpreting pooled results or making country-specific inferences.

Table 2 presents descriptive statistics for key study variables. The average age of participants was 49.04 years (SE = 0.05, 95% CI: 48.94–49.13). The mean score for depressed mood at baseline was 1.73 (SE < 0.01), increasing slightly to 1.75 at follow-up (95% CIs: 1.73–1.74 and 1.74–1.75, respectively). Similarly, the average anhedonia score increased from 1.84 at baseline to 1.86 at follow-up (SEs < 0.01; 95% CIs: 1.84–1.85 and 1.86–1.87, respectively). These small but consistent increases suggest a modest rise in depressive symptoms over the follow-up period.

Gender distribution was relatively balanced, with 51.09% identifying as female (SE = 0.14, 95% CI: 50.82–51.37) and 48.91% as male (SE = 0.14, 95% CI: 48.63–49.18).

Educational attainment varied, with 14.40% of the sample reporting low education levels (95% CI: 14.20–14.59), 54.93% reporting medium education (95% CI: 54.65–55.21), and 30.67% reporting high education (95% CI: 30.42–30.93).

More than half of the participants (57.83%) were married at baseline (SE = 0.14, 95% CI: 57.56–58.11), while 42.17% were not (SE = 0.14, 95% CI: 41.89–42.44).

Unemployment was relatively uncommon in the sample, with only 7.02% of participants unemployed at baseline (SE = 0.07, 95% CI: 6.88–7.17), compared to 92.98% who were not unemployed (95% CI: 92.83–93.12).

Regarding adverse childhood experiences (ACE), 14.11% of participants reported having experienced abuse (SE = 0.10, 95% CI: 13.92–14.31), while 85.89% did not (SE = 0.10, 95% CI: 85.69–86.08).

## 4. Structural Equation Modeling (SEM) in Females

**Figure 1:**
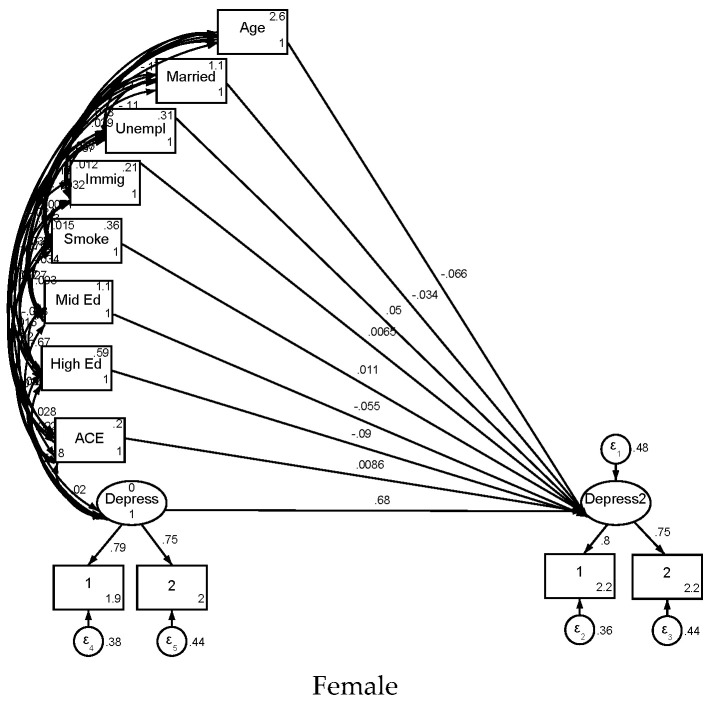

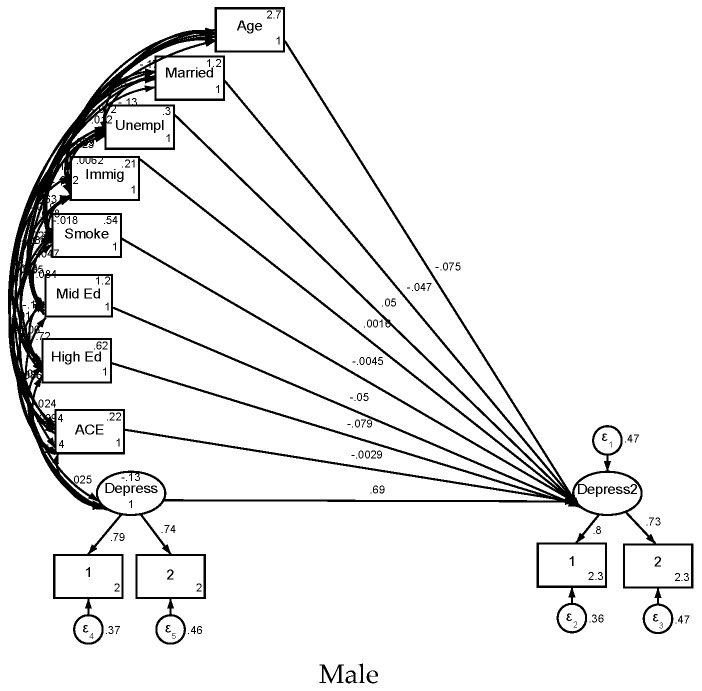
Summary of SEM in female and male participants.

Table 3 presents the SEM findings among females. ACE was significantly associated with a larger increase in depression (B = 0.009, *p* = 0.044). Immigration status did not show a statistically significant association (B = 0.006, *p* = 0.098). As expected, baseline depression was a strong predictor of follow-up depression (B = 0.677, *p* < 0.001) (Figure 1).

Higher educational attainment at baseline was significantly associated with fewer depressive symptoms at follow-up. Compared to women with low education, those with mid-level education had lower follow-up depression (B = −0.055, 95% CI: −0.066 to −0.045, *p* < 0.001), and the association was even stronger for those with high education (B = −0.090, 95% CI: −0.100 to −0.079, *p* < 0.001).

Other significant predictors of lower depression scores included older age (B = −0.066, *p* < 0.001) and being married at baseline (B = −0.034, *p* < 0.001). In contrast, unemployment (B = 0.050, *p* < 0.001) and smoking (B = 0.011, *p* = 0.011) were associated with higher depressive symptoms.

The measurement model showed high factor loadings for both depressed mood and anhedonia on the latent depression construct at baseline and follow-up. Depression strongly predicted both depressed mood (B = 0.800) and anhedonia (B = 0.749) at follow-up and baseline (B = 0.788 for depressed mood; B = 0.750 for anhedonia), all *p* < 0.001.

## 5. Structural Equation Modeling (SEM) in Males

As shown in Table 4, results among males were similar in many respects. However, ACE was not significantly associated with an increase in depression in males (B = −0.003, *p* = 0.524). Baseline depression remained the strongest predictor of follow-up depression (B = 0.688, *p* < 0.001).

Baseline educational attainment remained protective: mid-level education was associated with lower depression at follow-up (B = −0.050, 95% CI: −0.061 to −0.039, *p* < 0.001), and the effect was stronger for high education (B = −0.079, 95% CI: −0.090 to −0.068, *p* < 0.001).

Older age (B = −0.075, *p* < 0.001) and being married (B = −0.047, *p* < 0.001) were also associated with fewer depressive symptoms. Unemployment was linked to more depressive symptoms (B = 0.050, *p* < 0.001), while immigration status and smoking were not statistically significant (B = 0.002, *p* = 0.685 and B = −0.005, *p* = 0.279, respectively).

The measurement model also showed robust factor loadings: follow-up depression was strongly associated with both depressed mood (B = 0.801) and anhedonia (B = 0.730), and baseline depression similarly predicted its respective indicators (B = 0.794 for depressed mood; B = 0.735 for anhedonia), all *p* < 0.001.

## 6. Discussion

This longitudinal analysis of a large and diverse global sample highlights a key finding: childhood abuse is associated with increased depressive symptoms in adulthood among women, but not among men. For women, childhood abuse was associated with a 0.009-unit increase in depression scores (*p* = 0.044), whereas the effect was non-significant in men (B = −0.003, *p* = 0.524), indicating that women experienced, on average, a 1.2% greater increase in depressive symptoms compared to men. This observed sex/gender difference suggests a potentially unequal psychological burden of early-life adversity across sexes. While the overall link between childhood abuse and depression is well established, fewer studies have examined whether such adversity predicts changes in depression over time during adulthood, particularly in a global context.

The association between childhood abuse and greater depressive symptoms has been well documented in prior research [41,42,43]. Traumatic experiences early in life are thought to disrupt emotional development, heighten vulnerability to stress, and undermine long-term psychological well-being [44,45,46]. Within the framework of developmental psychopathology, adverse childhood environments are believed to interfere with the maturation of coping mechanisms and alter neurobiological systems involved in emotion regulation, such as stress-response pathways and mood-related brain circuits [47,48,49].

Our results are in line with many reviews [3,12] and empirical studies [13,14] on sex and gender differences in depression. Reviews highlight that the effects of ACEs on adult depression are modified by sex and associated biological mechanisms such as hormones, neurotransmission, the HPA axis, and epigenetics [22]. Such differences span prevalence, symptoms, treatment response, and neurobiology, including transcriptional patterns, immune signatures, and brain structure [12,14]. For instance, fluctuations in estrogen may amplify activation of the hypothalamic–pituitary–adrenal (HPA) axis in women, leading to heightened stress sensitivity following exposure to ACEs [50,51]. Additionally, societal norms that encourage women to internalize stress, in contrast to men who often adopt externalizing behaviors, may exacerbate vulnerability to depression [52].

However, unlike much of the existing literature that focuses on cross-sectional associations between ACEs and depression, our study specifically examined whether childhood abuse predicts future changes in depressive symptoms during adulthood. This focus on within-person change over time distinguishes our work from prior studies and adds new insight into the long-term emotional consequences of early adversity.

The sex/gender-specific pattern observed in our findings adds nuance to this literature [24,53]. Several plausible mechanisms may explain why women appear more vulnerable to the long-term mental health consequences of childhood abuse [54,55,56]. Biologically, sex hormones such as estrogen and progesterone influence the activity of the hypothalamic–pituitary–adrenal (HPA) axis, a central stress-regulation system [57,58]. These hormonal differences may amplify emotional reactivity and the internalization of distress among females following early trauma [59]. Neurodevelopmental studies have also shown sex-specific responses in brain regions critical to emotional regulation, such as the amygdala and prefrontal cortex, suggesting that early adversity may be encoded differently in male and female brains [60,61,62].

Sociocultural factors may further shape these divergent trajectories. Women are often socialized to express emotions inwardly and may be more likely to internalize stress and adversity, increasing the risk for depressive symptoms [63,64]. In contrast, men may be encouraged to suppress emotional expression or cope through externalizing behaviors, potentially masking the psychological impact of early trauma in typical depression scales [65,66,67]. Moreover, societal expectations and caregiving roles may increase chronic stress exposure for women, compounding the effects of childhood adversity [68,69]. Differences in coping styles may also play a role, as women are more likely to engage in rumination, a known risk factor for depression, while men may adopt distraction or avoidance strategies [70,71].

Together, these findings underscore the importance of considering sex and gender as central factors in understanding the long-term effects of childhood adversity. They also suggest that mental health interventions targeting the consequences of early trauma may need to be gender-sensitive to effectively address the distinct vulnerabilities and needs of women and men. This study adds to growing evidence that childhood adversity has long-term effects on mental health but highlights that these effects may not be experienced equally by men and women. By demonstrating a stronger association between childhood abuse and later depression among women, our findings point to the importance of sex/gender-sensitive approaches in both research and practice. Understanding and addressing these differences is crucial for reducing the lifelong burden of childhood trauma.

## 7. Policy and Practice Implications

Our findings have important implications for mental health policy and prevention. While childhood abuse should be prevented universally, our results suggest that particular attention is needed for girls, who may face more enduring psychological consequences. Mental health screening and trauma-informed interventions should be made available to adults with a history of childhood adversity—especially women—even many years after the original exposure. Sex/gender-responsive programming that acknowledges these disparities may improve outcomes and reduce the mental health burden associated with ACEs.

## 8. Strengths and Limitations

This study offers several notable strengths. The longitudinal design of the study allows for an examination of changes in depressive symptoms over time, rather than relying solely on cross-sectional associations. The international scope—with participants drawn from a diverse range of cultural, geographic, and socioeconomic backgrounds—adds important global context to the findings and increases their generalizability. The large sample size further strengthens the statistical power and precision of the estimates, enabling the detection of even modest effects and interactions, such as those observed by sex/gender.

Despite these strengths, several limitations warrant consideration. The uneven distribution of participants, with 25.58% from the USA, may affect the generalizability of findings, as cultural norms and healthcare systems in high-income countries may differ from those in low- or middle-income countries. Additionally, the lack of data on race, ethnicity, gender expression, or sexual orientation limits our ability to explore intersecting identities that may modify the impact of ACEs. Childhood abuse was assessed retrospectively using a single-item measure, which may be prone to recall bias or cultural interpretations of abuse, and provides limited information regarding the type, severity, frequency, and timing of abuse. This restricts our ability to explore dose–response relationships or to differentiate between forms of abuse (e.g., physical vs. emotional). The measure of depressive symptoms was also brief, based on only two self-reported items, which may not capture the full clinical range of depressive experiences and may be influenced by cross-cultural variation in emotional expression, mental health stigma, or help-seeking norms. Additionally, the follow-up period was relatively short—approximately one year—which may limit the ability to observe longer-term mental health trajectories following childhood adversity.

Our models were adjusted for core demographic and socioeconomic variables, but several important sources of heterogeneity were not included. Data on race, ethnicity, rural versus urban residence, gender expression, and sexual orientation were not available or were inconsistently collected across countries. These unmeasured factors may interact with both exposure and outcome, contributing to residual confounding or effect modification. Furthermore, the study focused only on childhood abuse and did not assess other types of adverse childhood experiences (ACEs) such as emotional neglect, household dysfunction, parental incarceration, or community violence, which are also known to impact adult mental health. The exclusion of these variables may underestimate the broader impact of early adversity and obscure potential cumulative or synergistic effects.

## 9. Future Research Directions

Future research should build on these findings by incorporating more detailed assessments of childhood adversity, including different forms of abuse, neglect, and household dysfunction, to better understand the cumulative and specific effects of adverse experiences. Prospective longitudinal studies, where possible, would help reduce the limitations of retrospective recall and provide a clearer temporal sequence between adversity and later outcomes. In addition, more work is needed to uncover the mechanisms behind the observed sex/gender differences, including investigations of biological stress systems, neuroendocrine functioning, and sexualized/gendered patterns of emotion regulation and socialization. Research should also explore how intersecting identities—such as race, ethnicity, class, gender expression, and sexual orientation—may compound or buffer the effects of ACEs on mental health. Future research may incorporate a more gender-inclusive perspective, recognizing the limitations of binary categorization. In addition, future sensitivity analyses ought to assess the robustness of the results against possible memory biases or underreporting in men. Future studies expanding participation in countries beyond the oversampled Western cultures would help to increase the generalizability of findings. Finally, future studies could evaluate the effectiveness of trauma-informed and sex/gender-responsive interventions across different cultural and health system contexts to identify strategies that are most effective in globally reducing the long-term mental health consequences of childhood abuse.

## 10. Conclusions

This study underscores the need for gender-sensitive mental health interventions addressing the greater vulnerability of women to the long-term consequences of childhood abuse. Policymakers should prioritize prevention and screening programs targeting adults with a history of ACEs, particularly women, while future research should focus on non-Western countries to explore cultural differences in the impact of ACEs and evaluate the effectiveness of tailored interventions.

## Figures and Tables

**Table 1:** Number and percentage of participants across countries.

Country	*n*	%	SE	95%	CI
Argentina	2884	2.31	0.04	2.23	2.39
Australia	2557	2.05	0.04	1.97	2.13
Brazil	4196	3.36	0.05	3.26	3.46
Egypt	3028	2.42	0.04	2.34	2.51
Germany	5476	4.38	0.06	4.27	4.50
India	6023	4.82	0.06	4.70	4.94
Indonesia	2664	2.13	0.04	2.05	2.21
Japan	13,841	11.07	0.09	10.90	11.25
Kenya	7660	6.13	0.07	6.00	6.26
Mexico	2229	1.78	0.04	1.71	1.86
Nigeria	3116	2.49	0.04	2.41	2.58
Philippines	2677	2.14	0.04	2.06	2.22
Poland	6419	5.14	0.06	5.01	5.26
South Africa	967	0.77	0.02	0.73	0.82
Spain	2880	2.30	0.04	2.22	2.39
Tanzania	5551	4.44	0.06	4.33	4.56
Türkiye	495	0.40	0.02	0.36	0.43
United Kingdom	3568	2.85	0.05	2.76	2.95
United States	31,970	25.58	0.12	25.34	25.82
Sweden	11,539	9.23	0.08	9.07	9.39
Hong Kong	704	0.56	0.02	0.52	0.61
China	4538	3.63	0.05	3.53	3.74

**Table 2:** Descriptive data.

	Mean	Std. err.	[95% conf.	Interval]
Age (Years)	49.04	0.05	48.94	49.13
Depressed Baseline	1.73	0.00	1.73	1.74
Depressed Follow Up	1.75	0.00	1.74	1.75
Anhedonia Baseline	1.84	0.00	1.84	1.85
Anhedonia Follow Up	1.86	0.00	1.86	1.87
Gender/Sex				
Female	51.09	0.14	50.82	51.37
Male	48.91	0.14	48.63	49.18
ACE				
No	85.89	0.10	85.69	86.08
Yes	14.11	0.10	13.92	14.31
Education				
Low	14.40	0.10	14.20	14.59
Mid	54.93	0.14	54.65	55.21
High	30.67	0.13	30.42	30.93
Married				
No	42.17	0.14	41.89	42.44
Yes	57.83	0.14	57.56	58.11
Unemployed				
No	92.98	0.07	92.83	93.12
Yes	7.02	0.07	6.88	7.17

**Table 3:** Summary of SEM in females.

		B	SE	95%	CI	*p*
Structural						
Depression (Follow Up)						
	Mid Education (Baseline)	−0.055	0.005	−0.066	−0.045	<0.001
	High Education (Baseline)	−0.090	0.005	−0.100	−0.079	<0.001
	Smoker (Baseline)	0.011	0.004	0.002	0.019	0.011
	Immigrant (Baseline)	0.006	0.004	−0.001	0.014	0.098
	Age (Yr) (Baseline)	−0.066	0.004	−0.073	−0.059	<0.001
	Unemployed (Baseline)	0.050	0.004	0.042	0.059	<0.001
	Married (Baseline)	−0.034	0.004	−0.042	−0.027	<0.001
	ACE	0.009	0.004	0.000	0.017	0.044
	Depression (Baseline)	0.677	0.004	0.669	0.684	<0.001
Measurement						
Depressed (Follow Up)						
	Depression (Follow Up)	0.800	0.003	0.795	0.805	<0.001
	Intercept	2.209	0.011	2.188	2.230	<0.001
Anhedonia (Follow Up)						
	Depression (Follow Up)	0.749	0.002	0.744	0.754	<0.001
	Intercept	2.237	0.010	2.217	2.258	<0.001
Depressed (Baseline)						
	Depression (Baseline)	0.788	0.002	0.784	0.793	<0.001
	Intercept	1.905	0.005	1.895	1.914	<0.001
Anhedonia (Baseline)						
	Depression (Baseline)	0.750	0.002	0.746	0.754	<0.001
	Intercept	1.952	0.005	1.942	1.962	<0.001

**Table 4:** Summary of SEM in males.

		B	SE	95%	CI	*p*
Structural						
Depression (Follow Up)						
	Mid Education (Baseline)	−0.050	0.006	−0.061	−0.039	<0.001
	High Education (Baseline)	−0.079	0.006	−0.090	−0.068	<0.001
	Smoker (Baseline)	−0.005	0.004	−0.013	0.004	0.279
	Immigrant (Baseline)	0.002	0.004	−0.006	0.009	0.685
	Age (Yr) (Baseline)	−0.075	0.004	−0.082	−0.067	<0.001
	Unemployed (Baseline)	0.050	0.004	0.042	0.059	<0.001
	Married (Baseline)	−0.047	0.004	−0.055	−0.039	<0.001
	ACE	−0.003	0.004	−0.012	0.006	0.524
	Depression (Baseline)	0.688	0.004	0.680	0.696	<0.001
Measurement						
Depressed (Follow Up)						
	Depression (Follow Up)	0.801	0.003	0.796	0.806	<0.001
	Intercept	2.295	0.011	2.274	2.317	<0.001
Anhedonia (Follow Up)						
	Depression (Follow Up)	0.730	0.003	0.725	0.735	<0.001
	Intercept	2.263	0.010	2.242	2.283	<0.001
Depressed (Baseline)						
	Depression (Baseline)	0.794	0.002	0.789	0.799	<0.001
	Intercept	1.976	0.005	1.965	1.986	<0.001
Anhedonia (Baseline)						
	Depression (Baseline)	0.735	0.002	0.731	0.740	<0.001
	Intercept	1.971	0.005	1.961	1.981	<0.001

## Data Availability

Data of the Global Flourishing Study ARE available through the Center for Open Science upon submission of a pre-registration (https://doi.org/10.17605/OSF.IO/3JTZ8) and will be openly available without pre-registration beginning 2026. Please see https://www.cos.io/gfs-access-data (accessed on 9 June 2025) for more information about data access.
